# IL-22 Protects against Biliary Ischemia-Reperfusion Injury after Liver Transplantation via Activating STAT3 and Reducing Apoptosis and Oxidative Stress Levels In Vitro and In Vivo

**DOI:** 10.1155/2022/9635075

**Published:** 2022-05-10

**Authors:** Yi Bai, Hao Wu, Jinrui Zhang, Sai Zhang, Zhixin Zhang, Hao Wang, Yamin Zhang, Zhongyang Shen

**Affiliations:** ^1^Department of Hepatobiliary Surgery, Tianjin First Central Hospital, School of Medicine, Nankai University, Tianjin, China; ^2^The First Central Clinical School, Tianjin Medical University, Tianjin, China; ^3^School of Medicine, Nankai University, Tianjin, China; ^4^Department of Organ Transplantation, Tianjin First Central Hospital, School of Medicine, Nankai University, Tianjin 300192, China; ^5^Key Laboratory of Transplant Medicine, Chinese Academy of Medical Sciences, Tianjin, China; ^6^National Health Commission's Key Laboratory for Critical Care Medicine, Tianjin, China

## Abstract

Biliary complications are currently one of the leading causes of liver failure and patient death after liver transplantation and need to be solved urgently. Biliary ischemia-reperfusion injury (IRI) is one of the important causes of biliary complications. IL-22 has a protective effect on liver injury and hepatitis diseases, and its safety and efficacy in the treatment of hepatitis have also been proved in human clinical experiments. Furthermore, multiple studies have confirmed that IL-22 promotes the proliferation and repair of epithelial cells in various organs. Still, its function in the bile duct after transplantation has not been explored. This study was aimed at investigating the effects of IL-22 on cholangiocyte IRI in vitro and in vivo and exploring its underlying mechanisms. We simulated the hypoxia process of bile duct epithelial cells through in vitro experiments to investigate the protective function and molecular mechanism of IL-22 on bile duct epithelial cells. Subsequently, the function and mechanism of IL-22 in the biliary IRI model of autologous orthotopic liver transplantation in rats were assessed. This study confirmed that IL-22 could promote cholangiocyte proliferation, decrease the apoptosis rate of cholangiocytes and tissues, decrease MDA levels, and increase SOD levels by activating STAT3. In addition, IL-22 can also reduce the level of mitochondrial membrane depolarization, protect mitochondria, reduce ROS production, and play a role in protecting bile ducts. These findings provide evidence for IL-22 as a novel and effective treatment for biliary IRI after liver transplantation.

## 1. Introduction

Currently, biliary tract complications are one of the primary causes of liver transplant failure and associated patient death, with an incidence of about 11% to 35% [1]. Compared with hepatocytes supplied by both portal vein and hepatic artery, bile duct epithelial cells only receive blood supply from the hepatic artery. Due to their diminished proliferation and repair capacity, they are more susceptible to ischemia-reperfusion injury (IRI) following transplantation [1]. The main manifestations of biliary complications after liver transplantation are necrosis and exfoliation of bile duct epithelial cells, bile duct fibrosis, and stricture, resulting in bile duct infection and obstruction. Cholestasis leads to impaired liver function; only a few patients can be successfully treated by endoscopic stents and drainage tubes or surgical treatment. However, most patients still lack safe and effective treatment measures [2–4]. Therefore, preventing and treating biliary complications after liver transplantation is an urgent clinical problem to be solved.

Interleukin-22 (IL-22) is a cytokine first cloned by Dumoutier et al. [5] in 2000. IL-22 is mainly secreted by immune cells, of which about 50% is secreted by T helper 22 cells (Th22), 33% by Th1 cells, and 15% by Th17 cells [6]. IL-22 receptor (IL-22R) is a heterodimer composed of IL-22 receptor 1 (IL-22R1) and IL-10 receptor 2 (IL-10R2). IL-10R2 is expressed in many cell types, while IL-22R1 is mainly expressed in epithelial cells such as the bronchi, liver, pancreas, and intestine. In contrast, it is hardly expressed in immune cells [7, 8]. Signal transducer and activator of transcription 3 (STAT3) is the primary mediator of IL-22 signal transduction. Phosphorylated STAT3 (p-STAT3) forms a homodimer for nuclear translocation and starts the transcription of downstream target genes as transcription factors. These target genes play an essential role in regulating cell growth, differentiation, and survival [9]. The protective role of IL-22 in liver injury and hepatitis disease has been widely studied. IL-22 treatment can activate the expression of many downstream genes, and these genes play an important role in acute-phase reaction, antiapoptosis and antioxidation, hepatocyte mitosis, and liver protection [10]. Our previous study also found that IL-22 can protect and promote liver regeneration by activating STAT3 after partial hepatectomy in the liver injury mouse model mediated by concanavalin A [11]. IL-22 can also reduce acute pancreatitis and intestinal mucosal injury in mice by activating the STAT3 signaling pathway and enhancing the expression of antimicrobial peptides and antiapoptotic genes [12].

Few studies have focused on biliary tract IRI after liver transplantation. In this study, the hypoxia process of bile duct epithelial cells was simulated in vitro, proving that IL-22 promotes the proliferation and repair of bile duct epithelial cells by activating the downstream STAT3 pathway, inhibiting IRI-induced apoptosis and reducing biliary complications. Subsequently, functional and mechanistic validation was performed in a rat autologous orthotopic liver transplantation biliary IRI model, aiming to provide a new option for clinical prevention and treatment of biliary complications after liver transplantation.

## 2. Materials and Methods

### 2.1. Cell Culture and Treatment

The human intrahepatic bile duct epithelial cells (HIBEpiCs) were purchased from ICell Bioscience Inc. (Shanghai, China). The human hepatocellular carcinoma cell line MHCC-97h and human mononuclear cell line THP-1 were obtained from the Cell Bank of the Chinese Academy of Sciences (Shanghai, China). The HIBEpiC and THP-1 were cultured in RPMI-1640 medium (Gibco, Grand Island, USA), and MHCC-97h was cultured in DMEM medium (Gibco, Grand Island, USA), all media containing 10% fetal bovine serum (Gibco, Grand Island, USA) and 1% penicillin/streptomycin (Beyotime, Shanghai, China). All three cell lines were grown in an incubator (37°C, 5% CO_2_).

Cobalt chloride (CoCl_2_, Sigma-Aldrich, USA) was used to simulate the hypoxia process of HIBEpiCs. The HIBEpiCs were divided into four groups: the control group (CON), the IL-22 (10 ng/ml) (Recombinant human IL-22, Absin, Shanghai, China) treatment group, the CoCl_2_ (150 *μ*M) treatment group, and the CoCl_2_ (150 *μ*M)+IL-22 (10 ng/ml) treatment group.

### 2.2. Animals and Groups

All adult male Sprague-Dawley (SD) rats (7~8 weeks old, 250~300 g) were purchased from Beijing Vital River Laboratory Animal Technology Co., Ltd. (Beijing, China). The animals were housed in a standard laboratory animal room with constant temperature (25°C approximately), at a humidity of 60%, 12 h/12 h light–dark cycle, and had free access to food and water. All animals received human care according to the standards outlined in the “Guidelines for the Care and Use of Laboratory Animals” prepared by the National Academy of Sciences, and all animal experiments were approved by the Ethics Committee of Tianjin First Central Hospital. The bile duct ischemia-reperfusion injury model of autologous orthotopic liver transplantation in rats was established based on previous studies [13]. A brief description of the surgical procedure is as follows: the first step is to free the perihepatic ligament and then ligate and cut off the left diaphragm vein, the right adrenal vein, and the pyloric branch of the portal vein. The suprahepatic and infrahepatic inferior vena cava was anatomized. The infrahepatic inferior vena cava, the abdominal aorta above the celiac trunk, and the distal end of the portal vein were occluded. The 2 ml (50 U/ml) heparin solution was pumped through the portal vein. The suprahepatic inferior vena cava was occluded. After that, the abdominal aorta was punctured, and heparin solution (25 U/ml) at 4°C was perfused. A hole of about 0.5 mm was cut above the blockage of the subhepatic cavity as the outflow tract. The suprahepatic inferior vena cava was clipped. After perfusion, the portal vein, hepatic artery, and inferior vena cava outflow tract were repaired. The portal vein and inferior vena cava were released from occlusion, and then, the hepatic artery was clamped for 30 minutes and then opened to establish a bile duct ischemia-reperfusion injury model. A total of 30 rats were divided into three equal groups. In the sham-operated group (*n* = 10), the rats underwent only laparotomy instead of autologous liver transplantation. In the IRI group (*n* = 10), rats were intraperitoneally injected with 0.9% normal saline at the same dose 2 hours before surgery, and then, the model was constructed. In contrast, rats (*n* = 10) were intraperitoneally injected with RcIL-22 (50 mg/kg, Supplementary Figure 1) 2 hours before surgery in the RcIL-22 (Recombinant Rat IL-22, Absin, Shanghai, China) treatment group. The rats were sacrificed 48 h after reperfusion, and the blood and bile duct tissues were collected for subsequent analysis.

### 2.3. RNA-seq and Differential Expression Analysis

Total RNA of CON group (*n* = 3) and IL-22 group (*n* = 3) of HIBEpiCs was extracted by Trizol (Invitrogen, USA). RNA integrity was assessed using the RNA Nano 6000 Assay Kit of the Bioanalyzer 2100 system (Agilent Technologies, CA, USA). Total RNA was used as input material for the RNA sample preparations. In order to preferentially select cDNA fragments of 370~420 bp, the library fragments were purified with the AMPure XP system (Beckman Coulter, Beverly, USA). Then, PCR was performed with Phusion High-Fidelity DNA polymerase, Universal PCR primers, and Index (X) Primer. Finally, the PCR products were purified (AMPure XP system), and the library quality was assessed with the Agilent Bioanalyzer 2100 system. The library preparations were sequenced on an Illumina NovaSeq platform, and 150 bp paired-end reads were generated.

Differential expression analysis of two groups was performed using the DESeq2 R package (1.20.0). The resulting *P* values were adjusted using the Benjamini and Hochberg's approach to control the false discovery rate. Genes with an adjusted *P* value < 0.05 found by DESeq2 were considered differentially expressed. The corrected *P* value and |log_2_ fold change| were set as the threshold for significant differential expression. Gene Ontology (GO) and Kyoto Encyclopedia of Genes and Genomes (KEGG) enrichment analyses of upregulated genes were performed among differentially expressed genes using clusterProfiler R package. A corrected *P* value less than 0.05 indicated significant enrichment.

### 2.4. Cell Viability

The cell viability was detected by the cell counting kit-8 (CCK-8, Boster, Wuhan, China) assay and calculated as the percentage of (OD test − OD blank)/(OD control − OD blank) [14]. Briefly, the cells were seeded into 96-well plates (1 × 10^4^ cells/well) for 24 h and then treated with different concentrations of CoCl_2_ or IL-22 at different times. After completing the treatment steps, 10 *μ*l CCK-8 was added to each well and incubated in an incubator (37°C, 5% CO_2_) for 1 h. Finally, the absorbance of each well was measured at 450 nm on a microplate reader (EnSpire, USA).

### 2.5. Wound-Healing Scratch Assay

The cells were seeded in advance in a 6-well dish. After the intervention, the cells were scraped with the tip of a yellow pipette and cultured in a serum-free medium for 48 h. The cultures were monitored and photographed at 6, 24, and 48 hours. ImageJ 7.0 software (National Institutes of Health, USA) was used to measure the distance between two edges of the wound surface.

### 2.6. Apoptosis Detection by Flow Cytometry

The Annexin-V-FITC/PI Apoptosis Detection Kit (KeyGEN, Nanjing, China) was used to detect cell apoptosis. After various interventions, the HIBEpiCs were digested with 0.25% EDTA-free trypsin (Boster, Wuhan, China) and collected. The cells were then resuspended in 500 *μ*l of binding buffer, 5 *μ*l of FITC-Annexin V, and 5 *μ*l of PI working solution and were added to each tube, which was incubated at room temperature for 15 minutes in the dark. Finally, flow cytometry (BD Accuri C6 Plus, Biosciences, USA) was carried out for detection. Apoptotic cells include early apoptotic cells and late apoptotic cells, and the data were processed using FlowJo V10.0 software.

### 2.7. Cell Cycle Detection by Flow Cytometry

The cell cycle was quantified by the Cell Cycle Detection Kit (KeyGEN, Nanjing, China). After the intervention, the cells were collected and fixed by adding 500 *μ*l of 70% cold ethanol and stored at 4°C overnight. The cells were collected by centrifugation, washed with PBS, and incubated at room temperature for 30 minutes with 500 *μ*l Rnase A/PI working solution. Finally, the cell cycle was detected by flow cytometry.

### 2.8. Measurement of Reactive Oxygen Species (ROS) Levels by Flow Cytometry

The intracellular ROS levels were detected using the reactive oxygen species assay kit (Solarbio, Beijing, China). Briefly, the cells were harvested and resuspended in a serum-free medium containing 10 mM DCFH-DA [14]. Subsequently, flow cytometry was performed to detect the DCF fluorescence intensity.

### 2.9. Measurement of Mitochondrial Membrane Potential by Flow Cytometry

The cell mitochondrial membrane potential was detected by the JC-1 mitochondrial membrane potential detection kit (Solarbio, Beijing, China). After collecting cells, 1 ml of JC-1 working solution was added, and the mixture was incubated at 37°C for 20 minutes. The cells were then washed twice with JC-1 buffer solution and added to the medium for flow cytometry.

### 2.10. Determination of Superoxide Dismutase (SOD) and Malondialdehyde (MDA)

The SOD and MDA levels of cells or tissues were detected using SOD and MDA detection kits (Beyotime, Shanghai, China). Firstly, the cell or tissue proteins were extracted and measured by BCA (KeyGEN, Nanjing, China) method. The corresponding working solution was added and left to react at 37°C for 30 minutes. Finally, a microplate reader was used to measure the absorbance of the corresponding wavelength and calculate the SOD and MDA levels according to the standard curve.

### 2.11. Histopathologic Evaluation

The bile duct tissues were fixed in 10% neutral formalin solution for 48 hours and then embedded in paraffin. The embedded tissues were sliced into 4 *μ*m thickness and stained with hematoxylin and eosin (HE). Human common bile duct tissue specimens were obtained from patients after pancreaticoduodenectomy. The patients had signed informed consent, and the experiment was approved by the ethics committee. Finally, the morphological changes of the bile duct tissues were observed under a microscope (Nikon, Japan). The severity of bile duct injury was assessed and scored according to the bile duct injury severity scale (BDISS, Supplementary Table 1) [15].

### 2.12. Immunohistochemical Staining

Briefly, the paraffin-embedded bile duct sections were deparaffinized using dimethylbenzene and hydrated with a gradient alcohol series. Then, the deparaffinized sections were treated with 3% H_2_O_2_ at room temperature for 30 min to block endogenous peroxidase activity. The sections were incubated with primary antibodies recognizing Cytokeratin-19 (CK-19, 1 : 300, Abcam, UK) and IL-22R (1 : 300, Abcam, UK) overnight at 4°C. Next, slides were washed with PBS and incubated with a biotinylated secondary antibody at room temperature for 1 hour. Finally, the cells with brown granules were observed as positive cells under the optical microscope.

### 2.13. Immunofluorescence Assays

The slides were submitted to a series of deparaffinization, hydration, antigen retrieval, and blocking steps as previously described. The slices were incubated with primary antibody Ki67 at 4°C overnight and then with isotype secondary antibody at room temperature for 1 hour. Finally, the sections were counterstained with DAPI and observed under a fluorescence microscope (Nikon, Japan).

### 2.14. Fluorescein TUNEL Staining

The apoptosis level of the bile duct was detected using a fluorescein TUNEL cell apoptosis detection kit (Servicebio, Wuhan, China). In brief, bile duct sections were treated with protease K (20 mg/ml) at 37°C for 20 min, then stained with TUNEL-FITC (1 : 200), and counterstained with DAPI at room temperature for 8 min. Finally, a fluorescence microscope was used for observation.

### 2.15. Biochemical Examination

The rats were sacrificed 48 hours after reperfusion, and serum samples were collected from the inferior vena cava for biochemical analysis. The levels of serum alanine aminotransferase (ALT), aspartate aminotransferase (AST), and total bilirubin (TBil) were detected by an automatic biochemical analyzer for animals (Mindray BS-240VET, USA).

### 2.16. Western Blotting

The total protein of cells and tissues was extracted using RIPA and PMSF mixed lysate (Solarbio, Beijing, China). The protein concentration was determined by the BCA method. Then, the proteins were separated by sodium dodecyl sulfate-polyacrylamide gel electrophoresis (SDS-PAGE, Boster, Wuhan, China). After the target proteins were transferred to the nitrocellulose filter (NC, Boster, Wuhan, China) membrane, it was blocked with 5% skimmed milk at room temperature for 1 hour. These membranes were incubated with primary antibodies such as IL-22R (1 : 1000, Proteintech, Wuhan, China), caspase3 (1 : 1000, Santa Cruz, USA), BCL2, (1 : 1000, Cell Signaling Technology, USA), BCLXL (1 : 1000, Cell Signaling Technology, USA), BAX (1 : 1000, Proteintech, Wuhan, China), HIF-1*α* (1 : 1000, Proteintech, Wuhan, China), *β*-actin (1 : 3000, Proteintech, Wuhan, China), GAPDH (1 : 1000, Santa Cruz, USA), STAT3 (1 : 2000, Cell Signaling Technology, USA), and p-STAT3 (Y705) (1 : 2000, Cell Signaling Technology, USA) overnight at 4°C. Subsequently, these membranes were washed 3 times with TBST buffer and incubated with the corresponding secondary antibody (1 : 3000) for 1 hour at room temperature. The membranes were exposed using an imaging system (Bio-Rad, Hercules, USA) and quantified using ImageJ 7.0 software.

### 2.17. Statistical Analysis

Data were analyzed using GraphPad Prism 8.0 statistical software (GraphPad Software, Inc., USA) and expressed as mean ± standard deviation (SD). Student's *t*-test was used to evaluate the significance between the two groups. A one-way analysis of variance (ANOVA) was applied to compare the differences among three or more groups, and the Tukey method was used for multiple comparisons between groups. *P* < 0.05 was considered to be statistically significant.

## 3. Results

### 3.1. IL-22 Reduces CoCl_2_-Induced Hypoxic Injury in IL-22 Receptor-Expressing Bile Duct Cells

**Figure 1:**
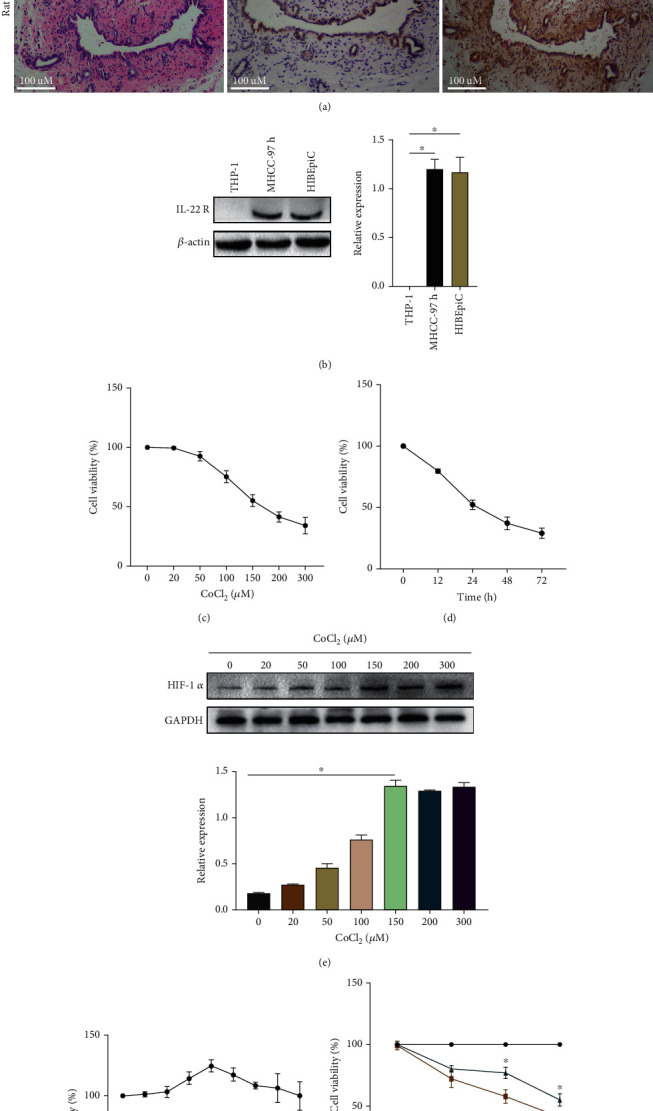
IL-22 receptor is expressed in bile duct tissue or cells, and IL-22 reduces CoCl_2_-induced cell damage in vitro. (a and b) Expression of IL-22R in human and rat bile duct tissues and HIBEpiCs. CK19: a marker of bile duct epithelial cells. THP-1 served as a negative control for IL-22R expression. MHCC-97h served as a positive control. (c) The inhibitory effect of CoCl_2_ on HIBEpiCs activity after being treated with 0~300 *μ*M for 24 h. (d) Cell viability at different time points treated with 150 *μ*M CoCl_2_. (e) HIF-1*α* protein expression with increasing CoCl_2_ concentration. (f) Viability of HIBEpiCs after treatment with 0~200 ng/ml IL-22 for 24 h. (g) The effect of 10 ng/ml IL-22 on 150 *μ*M CoCl_2_-induced HIBEpiCs injury at 24, 48, and 72 hours. Data are shown as mean ± SD. ^∗^*P* < 0.05.

In order to explore the effect of IL-22 on bile duct cells, the expression of IL-22 receptors in bile duct cells and tissues needs to be confirmed. Therefore, bile duct tissues were collected from rats and humans for immunohistochemical analysis. As shown in Figure 1(a), CK-19 (a biomarker of bile duct epithelial cells) and IL-22R were highly expressed in both human and rat bile duct tissues. In addition, proteins were extracted from THP-1 cells (negative control), MHCC-97h cells (positive control), and HIBEpiCs for Western blot analysis, and the results showed that HIBEpiCs also expressed IL-22R (Figure 1(b)). A cell hypoxia model was then induced by CoCl_2_. The results are displayed in Figures 1(c) and 1(d). As the CoCl_2_ concentration and exposure time increased, cell viability gradually decreased. After 24 hours of incubation at 150 *μ*m concentration, the cell viability was about 50%. The Western blot results demonstrated that the hypoxia marker protein HIF-1*α* also had the highest expression in HIBEpiCs treated with a CoCl_2_ concentration of 150uM (Figure 1(e)). To evaluate cytotoxicity, different concentrations of IL-22 (0~200 ng/ml) were set, and the viability of HIBEpiCs was detected by the CCK-8 method. As illustrated in Figure 1(f), IL-22 had no cytotoxic effect on HIBEpiCs and showed higher cell viability at 10 ng/ml. Further analysis showed a significant increase in cell viability after coculture with 10 ng/ml IL-22 for 48 and 72 h in HIBEpiCs stimulated by 150 *μ*M CoCl_2_ (*P* < 0.05, Figure 1(g)).

### 3.2. Differential Expression and GO/KEGG Enrichment Analysis

**Figure 2:**
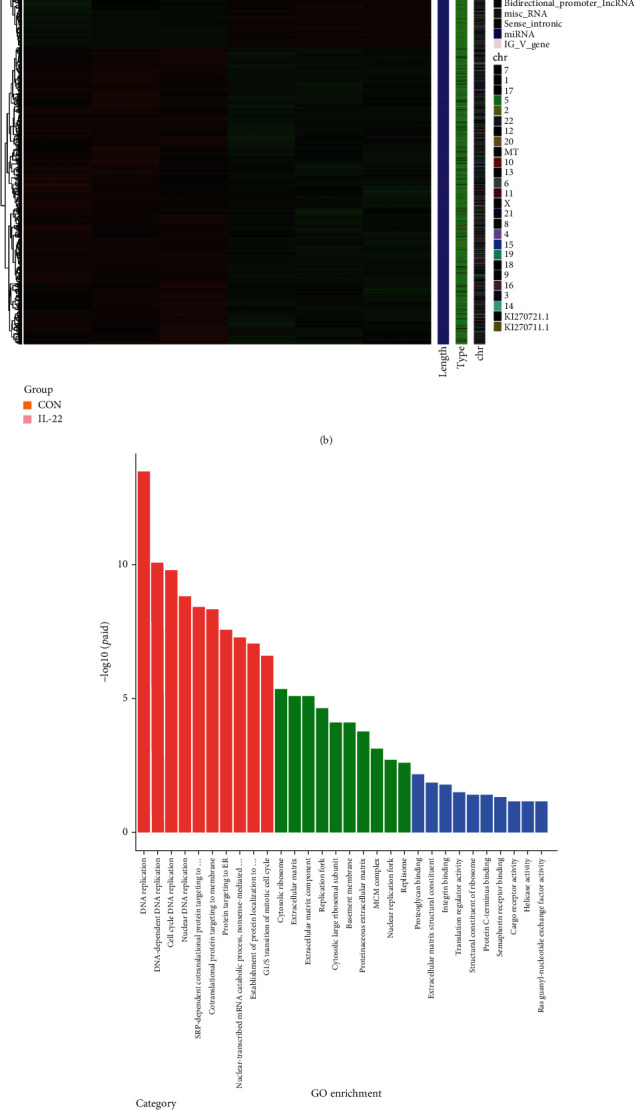
Differential expression and functional enrichment analysis. (a and b) Heat map and volcano map of differentially expressed genes in the IL-22 treatment group and CON group. (c) The GO functional enrichment analysis of upregulated genes after IL-22 treatment. (d) The KEGG enrichment analysis of upregulated genes after IL-22 treatment.

A total of 4901 differentially expressed genes (DEGs) were screened with |log_2_ fold change| ≥ 0.0 and *p*adj < 0.05, and 2277 of the 4901 DEGs were upregulated genes, while the remaining 2624 DEGs were downregulated genes (Figures 2(a) and 2(b)). Subsequently, GO and KEGG analyses were performed on the 2277 upregulated genes after IL-22 (10 ng/ml) treatment, revealing that the functions of these genes in biological processes were mainly related to DNA replication, cell cycle, and mitosis. The signaling pathways were also mainly enriched in DNA replication, mismatch repair, and cell cycle(Figures 2(c) and 2(d)). These results indicate that IL-22 can promote the upregulation of genes related to HIBEpiC proliferation.

### 3.3. IL-22 Promotes Proliferation and Migration of HIBEpiCs and Reverses CoCl_2_-Induced Proliferation Inhibition

**Figure 3:**
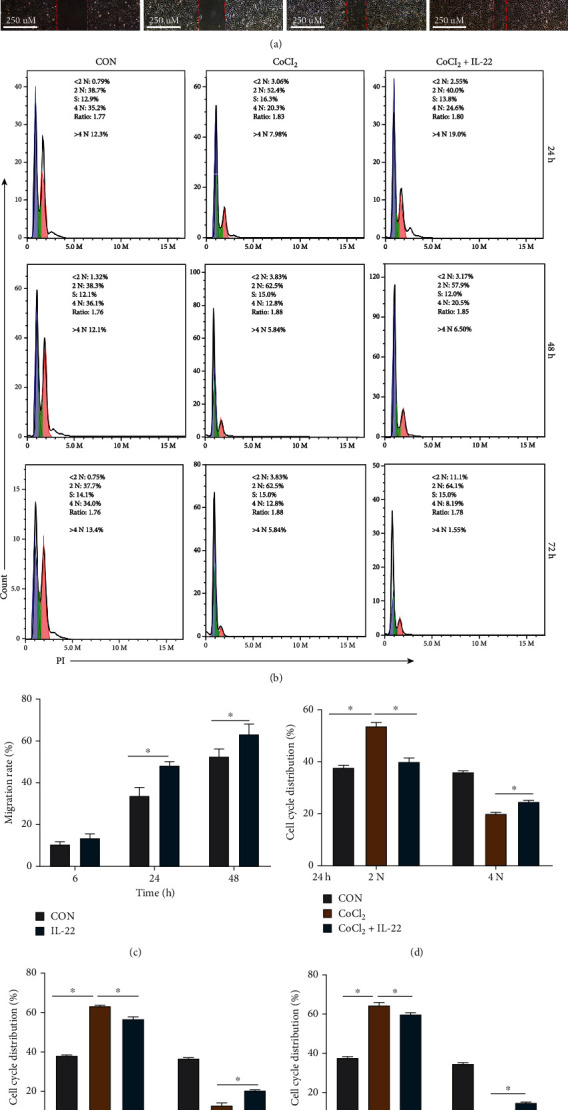
IL-22 can promote cell migration and proliferation in vitro. (a and c) Compared with the CON group, the cell migration rate was significantly increased at 24 and 48 hours after IL-22 treatment. (b and d~f) Compared with the CoCl_2_ group, the proportion of cells in the 4N phase in the CoCl_2_+IL-22 group was significantly increased at 24, 48, and 72 hours after IL-22 treatment. Data are shown as mean ± SD. ^∗^*P* < 0.05.

The effect of IL-22 on the migration ability of HIBEpiCs was detected by the scratch method. As shown in Figures 3(a) and 3(c), at the concentration of 10 ng/ml, IL-22 increased the migration ability of HIBEpiCs within 24 h and 48 h compared to the CON group (*P* < 0.05). At 6 h, there was no significant difference in migration rate between the IL-22 group and CON group for HIBEpiCs.

Flow cytometry was then used to detect the cell cycle of HIBEpiCs in the CON group, CoCl_2_, and CoCl_2_+IL-22 group. As displayed in Figures 3(b) and 3(d)–3(f), at 24, 48, and 72 h, compared with the CON group, the proportion of HIBEpiCs in the CoCl_2_ group increased in the 2N phase and decreased in the 4N phase (*P* < 0.05). Compared with the CoCl_2_ group, the proportion of HIBEpiCs in the CoCl_2_+IL-22 group decreased in the 2N phase, while the proportion in the 4N phase increased (*P* < 0.05). The results suggest that CoCl_2_ could arrest HIBEpiCs at the 2N phase, delaying the cell cycle progression and inhibiting cell proliferation. This process was reversed by IL-22, leading to a decrease in the proportion of HIBEpiCs in the 2N phase and an increase in the proportion in the 4N phase.

### 3.4. IL-22 Can Reduce CoCl_2_-Induced Apoptosis in HIBEpiCs In Vitro

**Figure 4:**
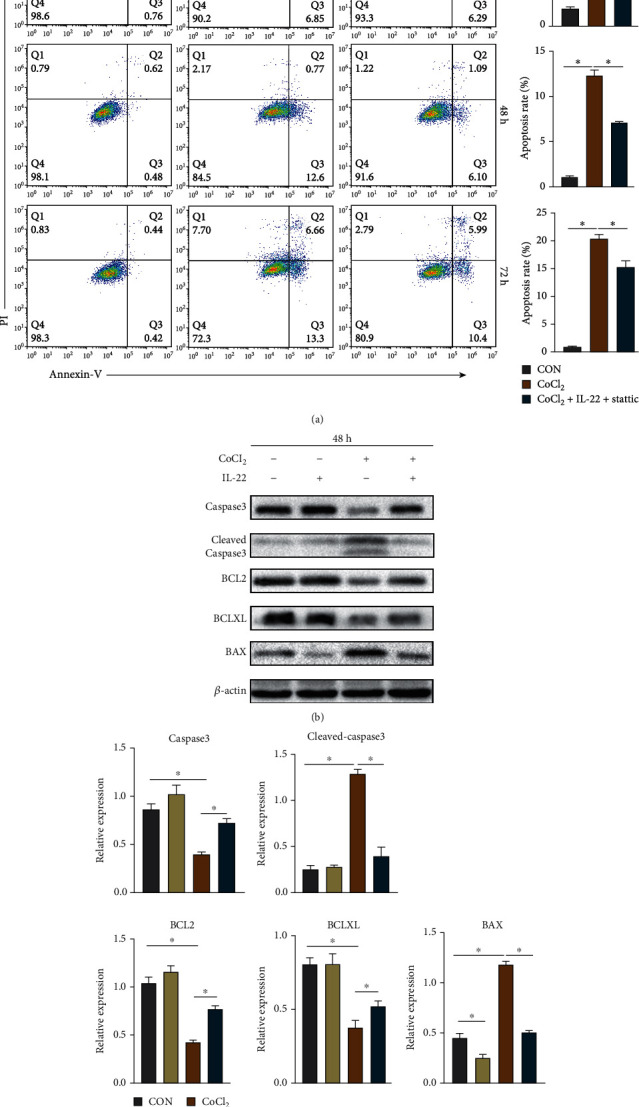
IL-22 can inhibit CoCl_2_-induced apoptosis of HIBEpiCs in vitro. (a) After treatment of CoCl_2_ alone and CoCl_2_+IL-22 combined treatment of HIBEpiCs for 24, 48, and 72 hours, the apoptosis level was detected by flow cytometry. IL-22 could significantly reduce the apoptosis level of HIBEpiCs at 48 and 72 hours. (b and c) Detection of apoptosis-related protein expression in different groups by Western blot assay. Data are shown as mean ± SD. ^∗^*P* < 0.05.

We cultured the HIBEpiCs of the CON group, CoCl_2_ group, and CoCl_2_+IL-22 group for 24, 48, and 72 h, respectively, then collected the cells, and detected the apoptosis level of each group by flow cytometry. As shown in Figure 4(a), compared with the CON group, the apoptosis rate of HIBEpiCs in the CoCl_2_ group was increased at 24, 48, and 72 h (*P* < 0.05). The apoptosis rate of the CoCl_2_+IL-22 group was lower than that of the CoCl_2_ group at 48 h and 72 h (*P* < 0.05). In addition, we extracted proteins from each group of cells at 48 h for Western blot assay, and the results showed that compared with the CON group, CoCl_2_ could significantly increase the expression of cleaved-caspase3 and BAX proapoptotic proteins and reduce the expression of BCL2 and BCLXL antiapoptotic proteins. In the CoCl_2_+IL-22 group, the expressions of cleaved-caspase3 and BAX proteins decreased, and the expressions of BCL2 and BCLXL proteins increased compared with the CoCl_2_ group (Figures 4(b) and 4(c)). The above results suggest that the protective effect of IL-22 against hypoxia may be related to its inhibition of apoptosis.

### 3.5. IL-22 Plays a Protective Role by Reducing Oxidative Stress Events in CoCl_2_-Induced HIBEpiCs Injury

**Figure 5:**
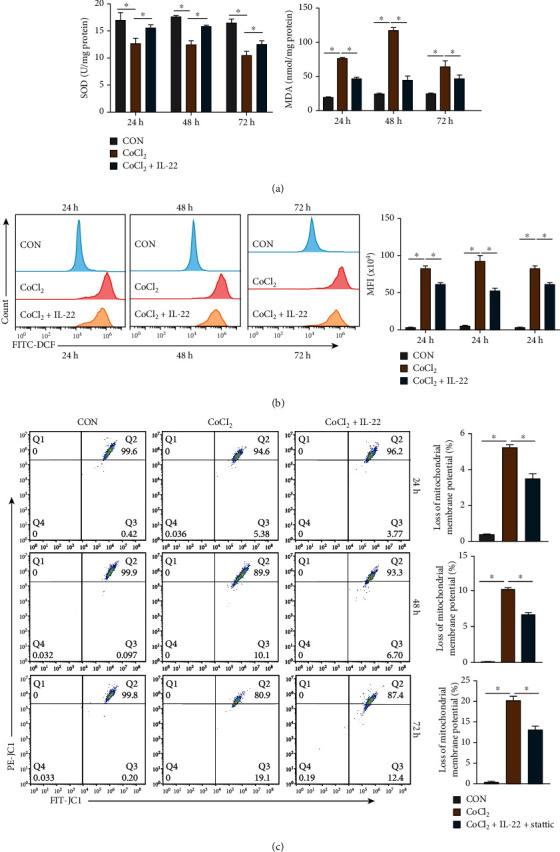
The antioxidative stress effects of IL-22 on CoCl_2_-induced HIBEpiC injury in vitro. (a) IL-22 could increase SOD levels and decrease MDA levels compared with the CoCl_2_ group. (b) Flow cytometry was used to detect the level of ROS in cells in each group. Compared with the CoCl_2_ group, IL-22 could significantly reduce ROS levels. (c) IL-22 can reduce the degree of mitochondrial membrane depolarization induced by CoCl_2_. Data are shown as mean ± SD. ^∗^*P* < 0.05. MFI: mean fluorescence intensity.

We detected the SOD, MDA, and ROS levels and mitochondrial membrane potential of HIBEpiCs in the CON, CoCl_2_, and CoCl_2_+IL-22 groups, respectively. Compared with the CON group, at 24, 48, and 72 h, lower SOD levels were found in CoCl_2_ group, while higher levels of MDA and ROS were detected. Furthermore, the proportion of depolarized cells increased in the CoCl_2_ group (*P* < 0.05). Compared with the CoCl_2_ group, the CoCl_2_+IL-22 group had higher SOD levels and lower levels of MDA and ROS, and the proportion of cells with decreased mitochondrial membrane potential was significantly reduced (*P* < 0.05, Figures 5(a)–5(c)). These results reveal that IL-22 can protect HIBEpiCs by reducing ROS activity and mitochondrial membrane depolarization.

### 3.6. IL-22 Inhibits Apoptosis and Reduces Oxidative Stress Levels by Activating STAT3

**Figure 6:**
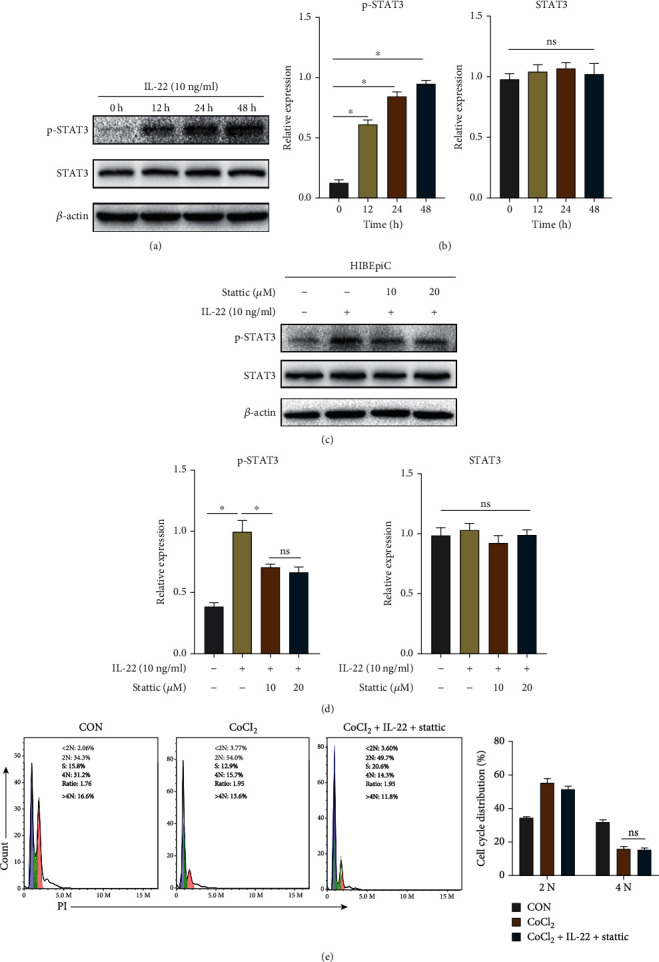
IL-22 plays a protective role by activating STAT3 in vitro. (a) Expression of STAT3 and p-STAT3 proteins in HIBEpiCs treated with IL-22 at different times. (b) Expression of STAT3 and p-STAT3 proteins after using 10 *μ*M and 20 *μ*M concentrations of STAT3 inhibitor stattic. (c) The cell cycle was detected after stattic (10 *μ*M) treatment, and there was no significant difference in the proportion of cells in the 4N phase compared with the CoCl_2_ group. Data are shown as mean ± SD. ^∗^*P* < 0.05. NS: no statistical difference.

Western blot experiments were carried out to explore the relationship between IL-22 and STAT3. The results are shown in Figures 6(a) and 6(b). Compared with the CON group, IL-22 treatment for 12, 24, and 48 h resulted in a significant increase in the p-STAT3 protein expression (*P* < 0.05). However, there was no significant difference in STAT3 protein expression among all groups. Subsequently, we treated the HIBEpiCs with the STAT3 inhibitor, stattic (MedChemExpress, USA), at concentrations of 10 *μ*M and 20 *μ*M for 48 h and detected the inhibition efficiency by western blot assay. The results are shown in Figures 6(c) and 6(d). At a stattic concentration of 10 or 20 *μ*M, the expression levels of p-STAT3 were all lower than the IL-22 group, so the 10 *μ*M concentration of stattic was chosen for follow-up studies.

**Figure 7:**
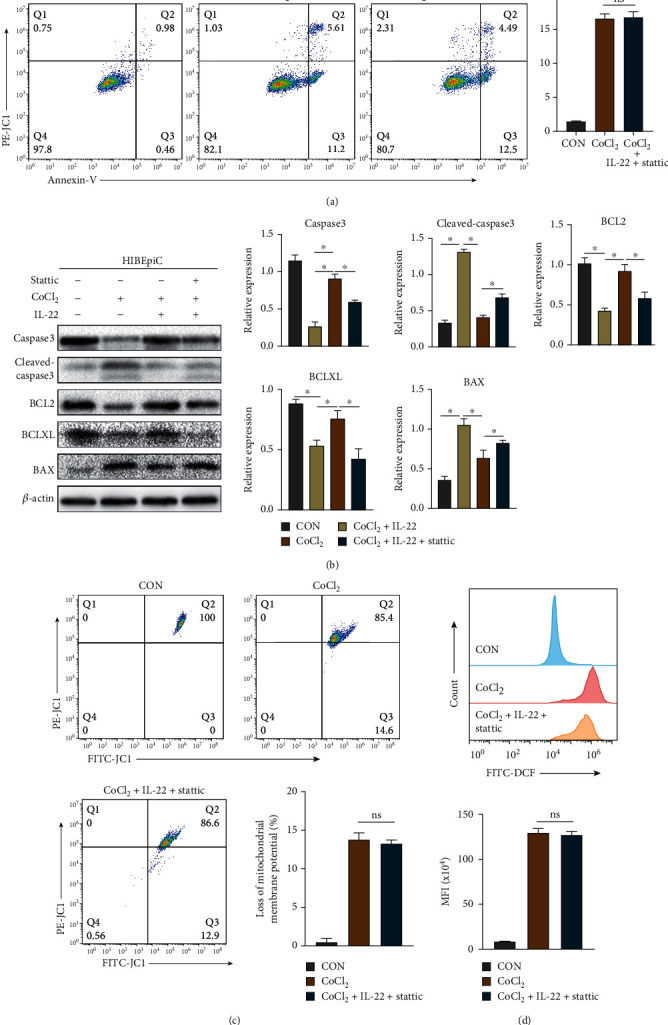
Effects of stattic treatment on apoptosis, ROS, and mitochondrial membrane potential in each group. (a) Apoptosis levels of HIBEpiCs in each group. There was no significant difference in apoptosis rate between stattic treatment and CoCl_2_ group. (b) After stattic treatment, the expression of antiapoptotic proteins decreased, and the expression of proapoptotic proteins increased compared with CoCl_2_+IL-22 group. (c) After stattic treatment, mitochondrial membrane potential was not significantly different from the CoCl_2_ group. (d) After stattic treatment, the level of ROS was not significantly different from the CoCl_2_ group. Data are shown as mean ± SD. ^∗^*P* < 0.05. NS: no statistical difference; MFI: mean fluorescence intensity.

We divided HIBEpiCs into the CON group, CoCl_2_ group, and CoCl_2_+IL-22+stattic group and cultured for 48 h, respectively. Then, the cell cycle, apoptosis, ROS, and mitochondrial membrane potential were detected by flow cytometry. As displayed in Figure 6(e), compared with the CoCl_2_ group, the cells in the CoCl_2_+IL-22+stattic group demonstrated no significant difference in the 4N phase (*P* > 0.05), indicating a decrease in the proportion of cells in the proliferation phase. In addition, the apoptosis level of HIBEpiCs in the CoCl_2_+IL-22+stattic group was not significantly different from that of the CoCl_2_ group. In contrast, higher expression levels of proapoptotic proteins BAX and cleaved-caspase3 were detected in the stattic-treated group compared with the CoCl_2_+IL-22 group, and lower expression levels of antiapoptotic proteins BCL2 and BCLXL were found (Figure 7(a) and 7(b)). Compared with the CoCl_2_ group, the CoCl_2_+IL-22+stattic group had no significant difference in ROS levels and the proportion of depolarized mitochondrial membrane cells (*P* > 0.05, Figures 7(c) and 7(d)).

### 3.7. IL-22 Reduces Apoptosis and Promotes Bile Duct Proliferation by Activating STAT3 in Rats

**Figure 8:**
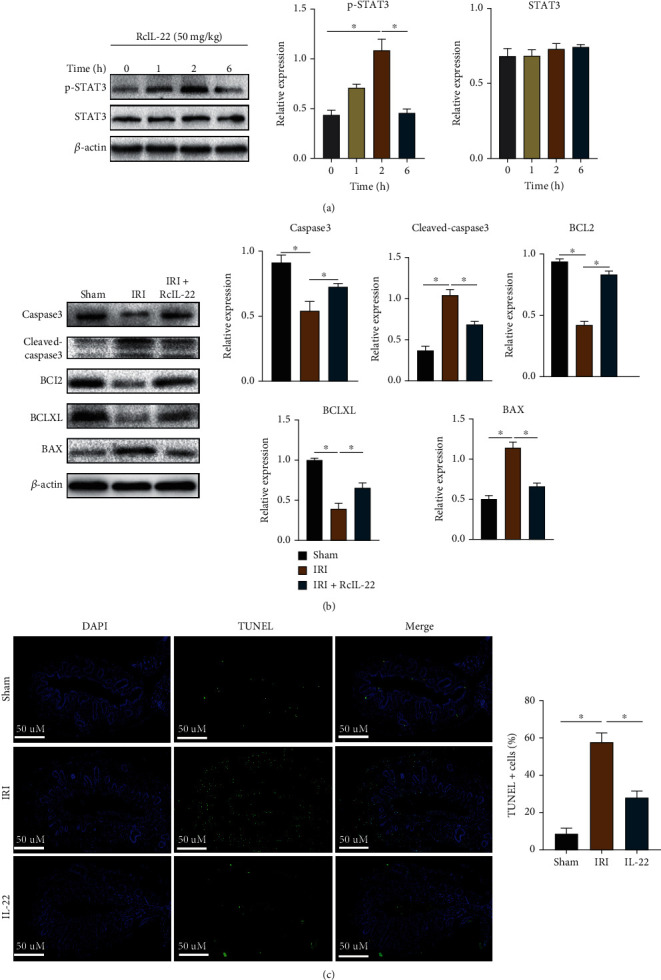
In vivo validation that IL-22 reduces IRI-induced apoptosis by activating STAT3. (a) The expression of STAT3 and p-STAT3 after intraperitoneal injection of RcIL-22 in rats for 1, 2, and 6 hours. (b) RcIL-22 can reduce the expression of cleaved-caspase3 and BAX protein and increase the expression of BCL2 and BCLXL protein in rats compared with the IRI group. (c) Evaluation of TUNEL expression in rat bile duct tissue sections by immunofluorescence staining. Data are shown as mean ± SD. ^∗^*P* < 0.05.

**Figure 9:**
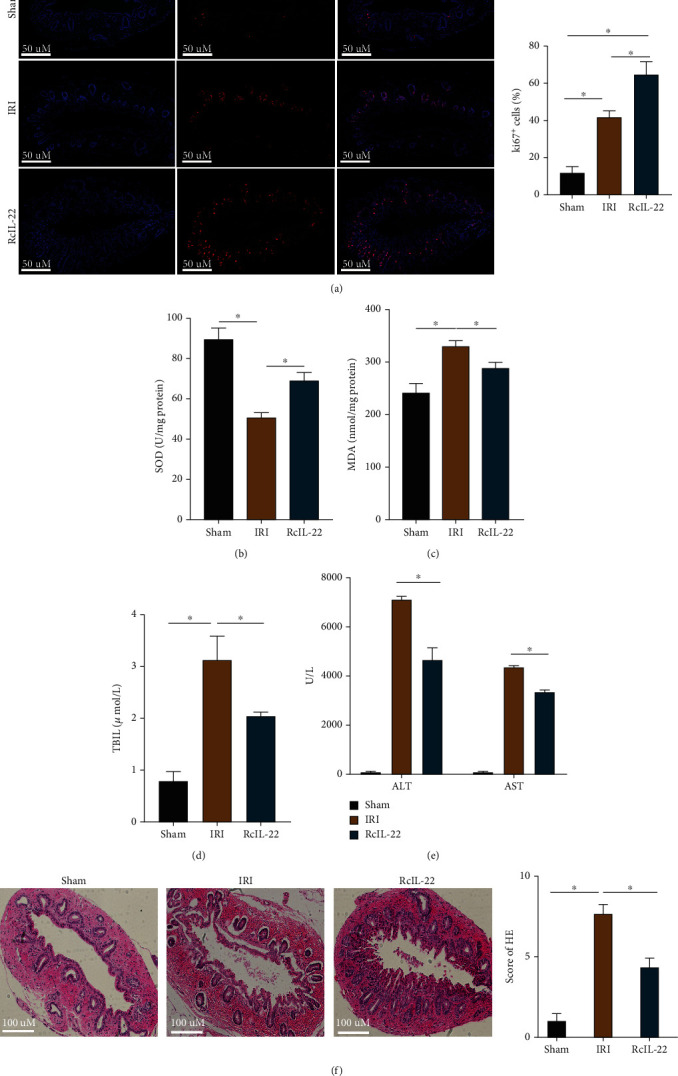
RcIL-22 promotes bile duct cell proliferation, reduces bile duct injury, improves liver function, and reduces bile duct oxidative stress levels in vivo. (a) The expression of Ki67 in rat bile duct tissue sections was evaluated by immunofluorescence staining, and the expression of Ki67 was higher after RCIL-22 treatment than in the IRI group. (b and c) RcIL-22 reduces MDA levels and increases SOD levels in rat bile duct tissue. (d and e) RcIL-22 reduced AST, ALT, and TBIL levels compared with the IRI group. (f) RcIL-22 can reduce the histological injury of the bile duct in rats induced by IRI. Data are shown as mean ± SD. ^∗^*P* < 0.05.

50 mg/kg RcIL-22 was injected into rats intraperitoneally, and the bile duct tissues were collected 1, 2, and 6 hours after injection for western blot assay. As shown in Figure 8(a), the expression of p-STAT3 protein increased 1 hour after injection, peaked at 2 hours, and decreased at 6 hours. Therefore, we chose to inject RcIL-22 2 hours before surgery. The animals were divided into sham group, IRI group, and IRI+RcIL-22 group, and bile duct tissues were collected 48 hours after reperfusion. As displayed in Figure 8(b), compared with the sham group, the expressions of the proapoptosis-related proteins cleaved-caspase3 and BAX increased in the IRI group, while the expressions of the antiapoptosis-related proteins BCL2 and BCLXL decreased. However, the expressions of cleaved-caspase3 and BAX in the IRI+RcIL-22 group were lower than those in the IRI group, and the expressions of BCL2 and BCLXL were higher (*P* < 0.05). The results of the TUNEL experiment revealed that IRI significantly induced apoptosis, while IL-22 could inhibit the induced apoptosis (*P* < 0.05, Figure 8(c)). In addition, tissue immunofluorescence results demonstrated increased Ki67 expression in IRI, and the use of RcIL-22 significantly increased the expression of Ki67 compared with the IRI group (*P* < 0.05, Figure 9(a)). The above results suggest that IL-22 can inhibit apoptosis and promote proliferation in vivo by activating STAT3.

### 3.8. IL-22 Reduces Oxidative Stress Levels and Alleviates Bile Duct Injury in Rats

The results are shown in Figures 9(b) and 9(c). Compared with the sham group, increased MDA levels and decreased SOD levels were detected in the IRI group (*P* < 0.05). Compared with the IRI group, RcIL-22 led to a significant decrease in MDA levels and increase in SOD levels (*P* < 0.05). Furthermore, RcIL-22 significantly reduced the levels of ALT, AST, and TBIL in rats (Figures 9(d) and 9(e)). On histological examination, severe bile duct injury was recorded in the IRI group. The bile duct epithelium was discontinuous, shed into the lumen, and infiltrated by inflammatory cells around the bile duct. However, the degree of bile duct injury in the RcIL-22 group was less severe than that in the IRI group, and the injury score was lower than that in the IRI group (*P* < 0.05, Figure 9(f)).

## 4. Discussion

Liver transplantation is the most effective treatment for end-stage liver disease [16]. However, biliary tract complications are serious complications following liver transplantation and significantly impact the long-term survival of liver transplantation patients. Ischemia-reperfusion injury of the bile duct after liver transplantation is one of the major causes of biliary complications [1]. Therefore, it is essential to study how to alleviate biliary ischemia-reperfusion injury. IL-22 has been confirmed to have various protective effects, such as promoting liver regeneration and antiapoptosis, promoting cell proliferation, and reducing the inflammatory response. Still, these effects have not been proved in bile ducts [10, 17, 18]. Therefore, this study intends to investigate the protective effect and mechanism of IL-22 on the bile duct through in vivo and in vitro experiments. The results confirmed that IL-22 could alleviate ischemia-reperfusion injury of bile ducts by activating STAT3 to inhibit apoptosis and reduce oxidative stress levels.

In this study, we first confirmed the high expression of IL-22R in human and rat bile ducts by immunohistochemistry, proving that IL-22 can exert its effects through its bile duct tissue IL-22 receptor. In addition, the expression of IL-22R in HIBEpiCs was detected and confirmed that IL-22R is not expressed in immune cells, indicating that IL-22 could have minimal side effects if used as treatment. It is known that CoCl_2_ can induce chemical hypoxia and is ideal for establishing the cellular hypoxia model [19]. Subsequently, CoCl_2_ was used to simulate the hypoxic state of cells in vitro, and the cell viability was detected by the CCK-8 method. The results confirmed that IL-22 could reverse the proliferation inhibition and damage caused by CoCl_2_ and exert a protective effect on HIBEpiCs.

In order to further explore the protective mechanism of IL-22 on HIBEpiCs, transcriptome sequencing and gene differential expression analysis were performed. Then, GO and KEGG enrichment analyses of upregulated genes after IL-22 treatment were carried out. The results revealed that the upregulated genes were mainly enriched in DNA replication and mitosis. In addition, cell scratch experiments also showed that IL-22 could promote HIBEpiC migration, further confirming that IL-22 can promote HIBEpiC proliferation. We then used flow cytometry to analyze the cell cycle of each group. The results showed that IL-22 could reverse the cycle arrest induced by CoCl_2_, resulting in an increase in the proportion of cells in the 4N phase. This indicates an increase in the proportion of cells in the proliferation phase. In addition, the bile ducts of rats showed an increase in compensatory Ki67^+^ cells after IRI. The proportion of ki67^+^ cells demonstrated a further significant increase after RcIL-22 treatment compared to the IRI group. The above results indicate that IL-22 could promote the proliferation of cholangiocytes both in vivo and in vitro.

Apoptosis is a form of programmed cell death that plays a vital role in immune system regulation, homeostasis, infection, damage, and clearance of senescent cells [20, 21]. The BCL2 antiapoptotic protein is a major regulator of the BCL2 family, which acts on the mitochondrial outer membrane to reduce the release of cytochrome C, thereby inhibiting apoptosis [22]. BAX is an important regulator of BCL2 activity, which can increase the permeability of the mitochondrial membrane, promote the release of cytochrome C, and activate the caspase cascade to promote apoptosis [23]. BCLXL is also a member of the BCL2 family and plays a role in inhibiting apoptosis [24]. Under hypoxic conditions, cells often undergo apoptosis [25, 26]. In this study, we detected the degree of apoptosis and the expression levels of apoptosis-related proteins in vitro and in vivo. We found that IL-22 could reduce the apoptosis induced by CoCl_2_ in vitro and reduce the apoptosis induced by IRI in vivo. In addition, IL-22 can significantly reduce the expression of proapoptotic proteins cleaved-caspase3 and BAX and increase the expression of antiapoptotic proteins BCL2 and BCLXL. These findings suggest that the antihypoxic effect of IL-22 may be related to its inhibitory effects on apoptosis.

Studies have shown that the inadequate ability of cells to deal with oxidative stress is usually manifested by a sharp increase in intracellular ROS and MDA levels and a decrease in SOD levels [27, 28]. MDA is a lipid peroxide that can promote the production of reactive oxygen species and cause tissue cell damage, and its level indirectly reflects the degree of cell damage. SOD is one of the key antioxidant enzymes in the body as it scavenges reactive oxygen species. Its level can reflect the antioxidant capacity of cells [29, 30]. Mitochondria provide cells with energy and play a crucial role in regulating cell survival. Permeability of the mitochondrial membrane is one of the triggering factors of apoptosis and necrosis. ROS disturbance leads to impaired mitochondrial inner membrane integrity and depolarization of the mitochondrial membrane. These changes further induce mitochondrial apoptosis and activate the caspase cascades [31, 32]. The current study found that IL-22 reduces MDA and ROS levels in vitro and increases mitochondrial membrane potential and SOD levels. In vivo experiments, IL-22 also reduces MDA levels and increases SOD levels in rat bile duct tissue. Furthermore, IL-22 can reduce bile duct injury and protect liver function in rats. These findings suggest that IL-22 exerts a protective role by reducing oxidative stress.

STAT3 is a key factor in the activation of the IL-22 signaling pathway. To verify the role of STAT3 in the IL-22 signaling pathway, we detected the expressions of STAT3 and p-STAT3 in vivo and in vitro, respectively. After intervention with IL-22, the expression of p-STAT3 increased, but there was no significant difference in the expression of STAT3, indicating that IL-22 could activate STAT3 and promote the effects of phosphorylated-STAT3. Stattic is an inhibitor of STAT3 activity [33], and decreased p-STAT3 expression levels were found after stattic treatment. Moreover, we detected the levels of apoptosis, cell cycle, ROS, and mitochondrial membrane potential in vitro. The findings revealed no significant differences in these indicators after the use of stattic compared with the hypoxia model group. The above results indicate that IL-22 exerts a protective effect by activating STAT3.

## 5. Conclusion

In summary, we simulated the hypoxia process of bile duct epithelial cells through in vitro cell experiments to investigate the protective effect of IL-22 on bile duct epithelial cells and its underlying molecular mechanism. The function and mechanism were verified in a rat autologous orthotopic liver transplantation biliary tract IRI model. We have identified for the first time that IL-22 reduces oxidative stress and apoptosis by activating STAT3 and promotes the proliferation of bile duct cells, thereby reducing the IRI injury of the bile duct and providing a new treatment option for treatment of bile duct complications after liver transplantation.

## Data Availability

The data of this study are available from the corresponding author upon reasonable request.

## Supplementary Materials

Supplementary Materials Table 1: bile duct injury severity scale. Figure 1: determination of RcIL-22 concentration.
