# Supplementary material for: IL-22 Protects against Biliary Ischemia-Reperfusion Injury after Liver Transplantation via Activating STAT3 and Reducing Apoptosis and Oxidative Stress Levels In Vitro and In Vivo
Source: Oxid Med Cell Longev. 2022 May 10;2022:9635075. doi: 10.1155/2022/9635075 (PMC9113870; doi:10.1155/2022/9635075)
Supplement: Supplementary Materials — Table 1: bile duct injury severity scale. Figure 1: determination of RcIL-22 concentration. [file 9635075.f1.docx]

| Degree of damage | Injury of bile duct epithelial cells | Bile duct inflammatory cell infiltration | Injury of glands around the bile duct |
| --- | --- | --- | --- |
| No | 0 | 0 | 0 |
| Mild | 1 | 1 | 1 |
| Moderate | 2 | 2 | 2 |
| Severe | 3 | 3 | 3 |

Supplementary Table 1. Bile duct injury severity scale.

Supplementary Figure 1. Determination of RcIL-22 concentration.


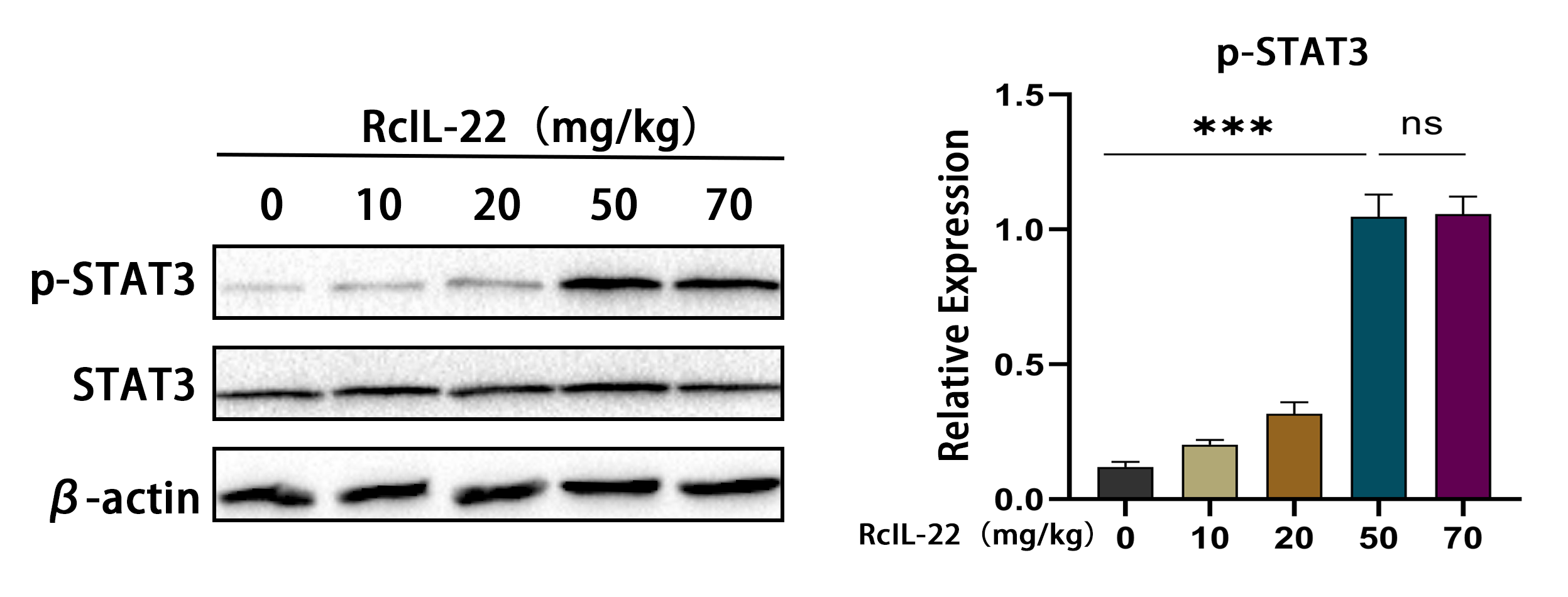


**Supplementary Figure 1**. As shown in the figure, when the concentration of RCIL-22 was 50 mg/kg, compared with the control group, the expression level of p-STAT3 was the highest (*p* < 0.01). When the concentration continued to increase, the expression level of p-STAT3 was not significantly different from before.
